# Nestin Forms a Flexible Cytoskeleton by Means of a Huge Tail Domain That Is Reversibly Stretched and Contracted by Weak Forces

**DOI:** 10.3390/cells14020138

**Published:** 2025-01-17

**Authors:** Ayana Yamagishi, Rina Tokuoka, Kazuki Imai, Mei Mizusawa, Moe Susaki, Koki Uchida, Saku T. Kijima, Akira Nagasaki, Daijiro Takeshita, Chiaki Yoshikawa, Taro Q. P. Uyeda, Chikashi Nakamura

**Affiliations:** 1 Cellular and Molecular Biotechnology Research Institute, National Institute of Advanced Industrial Science and Technology (AIST), Central 5 1-1-1 Higashi, Tsukuba 305-8565, Ibaraki, Japan; a-yamagishi@aist.go.jp (A.Y.);; 2 Department of Biotechnology and Life Science, Tokyo University of Agriculture and Technology, 2-24-16 Naka-cho, Koganei 184-8588, Tokyo, Japan; 3 Biomedical Research Institute, National Institute of Advanced Industrial Science and Technology (AIST), Central 6 1-1-1 Higashi, Tsukuba 305-8566, Ibaraki, Japan; 4 Research Center for Macromolecules and Biomaterials, National Institute for Materials Science, 1-2-1 Sengen, Tsukuba 305-0047, Ibaraki, Japan; 5 Department of Physics, Faculty of Science and Engineering, Waseda University, 3-4-1 Okubo, Shinjuku-ku, Tokyo 169-8555, Japan

**Keywords:** nestin, intermediate filament, actin, atomic force microscope

## Abstract

Nestin is a type VI intermediate filament protein and a well-known neural stem cell marker. It is also expressed in high-grade cancer cells, forming copolymerized filaments with vimentin. We previously showed that nestin inhibits the binding of vimentin’s tail domain to actin filaments (AFs) by steric hindrance through its large nestin tail domain (NTD), thereby increasing three-dimensional cytoskeleton network mobility, enhancing cell flexibility, and promoting cancer progression. Further, we found that nestin itself stably binds to AFs via the NTD. We therefore hypothesized that the NTD may form a flexible cytoskeletal structure by extending with weak force. In vitro tensile tests using atomic force microscopy were performed to assess the mechanical properties of NTDs. The C-terminus of the NTD bound AFs by bringing the AFM tip modified with the NTD into contact with the AFs on the substrate. NTDs were elongated to approximately 80% of their maximum length at weak forces < 150 pN. Repeated tensile tests revealed that the NTD refolded quickly and behaved like a soft elastic material. We speculate that nestin stably binds AFs, and the NTD extends with weak force, contracting quickly upon load release. Thereby, nestin would absorb mechanical load and maintain cytoskeletal integrity.

## 1. Introduction

The cytoskeleton is a three-dimensional network structure formed by the interconnections of actin filaments (AFs), microtubules, and intermediate filaments (IFs) [1]. IFs exist in the cytoplasm in a meshwork pattern and play important roles in maintaining cell morphology and elasticity. IF proteins are composed of head, rod, and tail domains from the N-terminal side. The rod domain forms a parallel coiled coil with another rod domain to form a dimer. A pair of parallel dimers then associates in an antiparallel fashion to form a tetramer. This tetramer is the soluble subunit and bundles to form a single filament [2].

Nestin, a type VI IF protein, is known as a marker for neural stem cells. Nestin has a characteristic large tail domain of 170 kDa and cannot form filaments on its own, requiring a partner such as vimentin [3]. The nestin tail domain (NTD), which protrudes from the filament surface, is thought to function as an integration platform for cell signals by interacting with various proteins. For example, NTD has been reported to have a number of functions, including inhibiting myogenic differentiation by inhibiting the generation of p25 through calpain-mediated p35 cleavage [4], promoting the formation of stress fibers by binding to Fhos2 [5], regulating the ssh pathway by binding to Gli3 [6], and promoting the generation of antioxidant enzymes by interacting with Keap1 to prevent Nrf2 from being degraded [7].

Nestin is overexpressed in highly malignant cancer cells. It expression is strongly associated with aggressive breast cancer types characterized by stem cell-like properties [8]. In ovarian cancer, high nestin expression correlates with a 0% five-year survival rate, underscoring its prognostic significance [9]. Similarly, reducing nestin expression in prostate cancer cells has been shown to decrease invasiveness [10]. Despite these associations, the molecular mechanisms underlying nestin’s role in cancer cell malignancy remain largely unclear, leaving a critical gap in understanding its contribution to cancer progression.

We have investigated the properties of nestin in highly metastatic mouse breast cancer cells [11]. Firstly, we investigated cellular elasticity and found that the stiffness of the nestin knockout cells was greater than that of parental cells. This suggests that nestin can make cells more easily deformable, facilitating their passage through the gaps between connective tissues to increase invasiveness and malignancy. This is consistent with previous findings showing that cancer cells are softer than normal cells [12]. To elucidate the mechanism through which nestin decrease cellular stiffness, we focused on its characteristic large NTD of 170 kDa. In the examined cancer cells, it forms copolymerized filaments with vimentin. Vimentin binds to actin filaments (AFs) via its tail domain [13,14], and we supposed that the binding of vimentin to AFs near the cell membrane contributes to cytoskeleton reinforcement and the maintenance of cell elasticity. When vimentin forms a dimer with nestin, their tail domains are in close proximity. Proximity ligation assays (PLAs) suggested that nestin inhibits vimentin–AF binding via steric hindrance, thereby weakening the cytoskeletal connectivity and softening cells [11]. In fact, when the mobility of the copolymerized filaments of vimentin and nestin was examined using an antibody-modified nanoneedle operated with an atomic force microscope system, it was shown that the mobility of filaments containing nestin was higher than that of those containing vimentin alone, and this would contribute to cytoskeletal flexibility and make the cell softer [11]. In other words, IF–AF binding contributes to cell stiffness. On the other hand, further PLA analyses suggest that NTDs and AFs may also bind each other. The formation of NTD–AF binding would lower cytoskeletal flexibility. Therefore, the aim of the current study was to determine whether the NTD binds to AFs and to consider the mechanical properties of the NTD.

## 2. Materials and Methods

### 2.1. Cell Culture

The highly malignant mouse breast cancer cell line FP10SC2 (SC2) was previously established from the parental cell line 4T1 (American Type Culture Collection; ATCC, Manassas, VA, USA) by Okada et al. [15]. The SC2 cells were cultured at 37 °C in a humidified atmosphere with 5% CO_2_ in RPMI 1640 medium (Sigma-Aldrich, St. Louis, MO, USA) supplemented with 2 mM l-glutamine (Wako Pure Chemical Industries, Ltd., Osaka, Japan), 1.5 g/L sodium bicarbonate (Invitrogen, Carlsbad, CA, USA), 2.5 g/L glucose (Wako), 10 mM HEPES pH 7.4 (Sigma-Aldrich), 1 mM sodium pyruvate (Wako), and 10% fetal bovine serum (FBS; Thermo Fisher Scientific, Inc., Waltham, MA, USA). The cells were treated with PBS containing 0.25% trypsin–EDTA (Thermo Fisher Scientific) and centrifuged to form a pellet. The resulting pellet was dispersed, and the cells were seeded into glass-bottom culture dishes (IWAKI, Tokyo, Japan). Human osteosarcoma U2OS cell line purchased from the RIKEN BioResource Research Center (Ibaraki, Japan) was cultured in Dulbecco’s Modified Eagle Medium (DMEM) (Sigma-Aldrich) containing 10% FBS under the same conditions as described above.

### 2.2. Proximity Ligation Assay

The proximity between nestin and AFs was examined using the Duolink in situ proximity ligation assay (Sigma-Aldrich). SC2 cells were cultured and fixed in 4% paraformaldehyde for 15 min. After permeabilization with cold acetone for 1 min, the cells were washed three times with PBS and treated with 0.4% Block Ace (KAC, Kyoto, Japan) in PBS for 1 h. The cells were then incubated with mouse monoclonal anti-nestin antibodies (1:1000; MAB353; Merck Millipore, Billerica, MA, USA) and rabbit monoclonal anti-actin (1:40; A2066; Sigma-Aldrich) diluted in 0.4% Block Ace for 1.5 h at room temperature. All other steps were performed according to manufacturer instructions. As a positive control, rabbit monoclonal anti-vimentin antibodies (1:500; EPR3776; Abcam, Cambridge, UK) were used to detect vimentin–AF binding in SC2 cells, and a nestin knockout cell line was used as a negative control [11]. Fluorescence microscopy was performed using IX-70 (Olympus, Tokyo, Japan) equipped with a DP30BW camera (Olympus, Japan).

### 2.3. Plasmid Construction

Mouse nestin and vimentin cDNAs were cloned from mouse cDNA synthesized using total RNA extracted from mouse brain. The pEGFP-NTD_1358–1864_ plasmid for GFP-NTD_1358–1864_ expression in SC2 and U2OS cells was constructed by cloning a PCR product amplified using the following primer pair: 5′-CTCGAGGGTGGCTCTGGAGGCT-3′ and 5′-AAGCTTGGTGGCGACCGGTGGA-3′. The PCR product was inserted between the *Xho*I and *Sal*I sites of pEGFP-C3 (Clontech, Tokyo, Japan).

A series of vectors for GFP-NTD proteins expressed in *E. coli* were generated by inserting a GFP gene fragment between the *Sac*I and *Kpn*I sites of pColdI (Takara). A series of NTD fragments were inserted between the *Hin*dIII and *Sal*I sites. By using pColdI, a His tag is added to the N-terminus of GFP-NTD. The Lifeact-EGFP expression vector was previously described [16].

For the whole NTD expression, pET-22b was modified to express the protein with a 6xHis and SUMO tag at the N terminus. The gene encoding NTD was cloned into the pET-22b-His-SUMO plasmid, and a 6×His-tag sequence was introduced between the SUMO and NTD genes using a PCR-based method.

Cloned gene sequences were confirmed by Sanger sequencing.

### 2.4. Microscopy of Colocalization

SC2 and U2OS cells were transfected with the vector pEGFP-NTD1358-1864, as described in Section 2.3, using Lipofectamine2000 (Thermo Fisher Scientific). For rhodamine phalloidin staining, the transfected cells on a glass-bottom dish were fixed with PBS containing 3.7% formaldehyde (Wako) for 30 min and were permeabilized with PBS containing 0.1% Triton X-100 for 10 min. The cells were then stained with 100 nM rhodamine-labeled phalloidin (Molecular Probes, Eugene, OR, USA) for 20 min at room temperature. Images were captured with an IX71 microscope (Olympus) equipped with a CSU10 confocal unit (Yokogawa Electric, Tokyo, Japan) and a dual-view system (Optical insights, BioVision Technologies, Inc., Exton, PA, USA). Z projection images were created with ImageJ software 1.53q (NIH, MD, USA).

### 2.5. Purification of Proteins

To prepare a series of GFP-NTD proteins, *Escherichia coli* strain BL21 (DE3) was transformed with the expression vectors described in Section 2.3. The transformant cells were cultured at 37 °C with shaking until the OD_600_ reached 0.5, then at 15 °C for 30 min with shaking. Protein expression was induced using 1 mM isopropyl β-D-1-thiogalactopyranoside (IPTG). After 16 h, the collected cells were resuspended in a lysis buffer (20 mM Tris-HCl and 100 mM NaCl, pH 7.5) and sonicated in the presence of a protease inhibitor cocktail (Wako). For protein purification, the supernatant obtained after centrifugation was added to Ni-nitrilotriacetic acid (NTA) agarose (QIAGEN, Netherlands), the resin was washed (using 20 mM Tris-HCl, 100 mM NaCl, and 10 mM imidazole, pH 8.0), and the recombinant protein was eluted with elution buffer (20 mM Tris-HCl, 100 mM NaCl, and 100 mM imidazole, pH 8.0). The eluate was further concentrated using an ultrafiltration device (Amicon Ultra-4 30 K; Merck) and PBS.

For His-tagged whole NTD preparation, *Escherichia coli* BL21(DE3) was transformed with the plasmid pET-SUMO-His-Nestin tail domain and cultured at 37 °C until the OD_600_ of the culture reached 0.8–1.0. Protein expression was induced by the addition of 0.1 mM IPTG. The collected cells were disrupted by sonication in lysis buffer (50 mM sodium phosphate, 300 mM NaCl, 5.0 mM β-mercaptoethanol (β-ME), 10% glycerol, and 10 mM imidazole, pH 8.0) and centrifuged. The supernatant was loaded onto a Ni-NTA agarose column (QIAGEN, Hilden, Germany) and washed with wash buffer (50 mM sodium phosphate, 300 mM NaCl, 5.0 mM β-ME, 10% glycerol, and 20 mM imidazole, pH 8.0). The protein was eluted with the same buffer containing 200 mM imidazole. The eluted pool was loaded onto a HiLoad 16/60 Superdex 200 pg column (Cytiva, Marlborough, MA, USA) in buffer consisting of 20 mM Tris-HCl (pH 8.0), 300 mM NaCl, 10% glycerol, and 5.0 mM β-ME, and the peak fractions were collected. The SUMO tag was removed by incubation with Ulp1 (SUMO protease) at 4 °C for 15 h. The mixture was loaded onto a HiLoad 16/60 Superdex 200 pg column (Cytiva), and the peak fractions were dialyzed against a buffer containing 20 mM Tris-HCl (pH 8.0), 100 mM NaCl, 10% glycerol, and 5.0 mM β-ME. The dialyzed sample was applied to a HiTrap Q column (Cytiva) and eluted using a linear gradient of NaCl from 100 mM to 1 M in the same buffer. Peak fractions of the His-tagged NTD were concentrated using an ultrafiltration device (Amicon Ultra-4 30 K; Merck).

### 2.6. Preparation of AF Substrate

Skeletal muscle G-actin was extracted from acetone powder of chicken breast muscle, purified using the method of Spudich and Watt [17], snap-frozen, and stored at −80 °C. Actin was allowed to polymerize at 4 µM in actin polymerization buffer (10 mM HEPES, 0.15 M KCl, 2 mM MgCl_2_, 0.2 mM ATP, and 1 mM DTT, pH 7.4) for 1 h at room temperature. The resulting 4 µM AFs were stained with rhodamine–phalloidin added to a final concentration of 0.4 µM. For the immobilization of AFs, glass coverslips or 27φ glass-base dishes were treated with 1 mM aminopropyltriethoxysilane (APTES) in 99.5% ethanol and incubated for 1 h at room temperature. After washing three times with ethanol and drying up, 10 µL of 20 nM rhodamine–phalloidin-stained AFs in actin polymerization buffer was incubated for 30 min at room temperature in a humid environment for in vitro interaction analysis between GFP-NTD and AFs. After washing the unbound AFs with the actin polymerization buffer, the glass surface was incubated with 10 mg/mL bovine serum albumin (BSA) in the actin polymerization buffer for 30 min at room temperature.

For tensile tests of the NTD, immobilization of the AFs to the glass substrate with covalent bonding was added to the above protocol after the APTES modification step. Firstly, 10% glutaraldehyde in ethanol was added to an APTES-modified 27φ glass-base dish and incubated for 1 h. After washing three times with ethanol, the dish was washed once with actin polymerization buffer. Ten microliters of 20 nM rhodamine–phalloidin-stained AFs was added and incubated for 30 min at room temperature. Subsequently, the surface was washed three times with 1 mL of actin polymerization buffer for 5 min. Finally, BSA was used to block aldehyde groups to which actin was not bound. A 130 mg/mL BSA solution dissolved in actin polymerization buffer was added dropwise and allowed to stand for 30 min at room temperature. Subsequently, the dish was washed three times with an actin polymerization buffer for 5 min each.

### 2.7. In Vitro Interaction Analysis Between AFs and Nestin

Ten microliters of actin polymerization buffer containing 0.2 mg/mL GFP-Q1~Q4NTD, GFP-Lifeact, or GFP protein diluted to 0.2 mg/mL in actin polymerization buffer was added to the AF-immobilized substrate and incubated for 30 min at room temperature. After 10 washes with high-salt actin polymerization buffer (10 mM HEPES pH 7.4, 300 mM KCl, 2 mM MgCl_2_, 0.2 mM ATP, and 1 mM DTT), the samples were observed under a fluorescence microscope (IX-70) equipped with a CMOS camera (ORCA-Flash 2.8; Hamamatsu Photonics, Hamamatsu, Japan). The intensity of green fluorescence on AFs and the background was evaluated using ImageJ software.

### 2.8. Modification of the Nestin Tail on a Cantilever

In this study, a terpolymer produced by radical polymerization of dopamine methacrylamide (DMA)/2-hydroxypropyl acrylamide (HPA)/*N*-succinimidyl methacrylate (NHS) was used to immobilize proteins on silicon atomic force microscopy (AFM) cantilevers [18]. DMA is used as a functional group that adheres to the silicon oxide surface, imitating mussel adhesion to rocks via the catechol groups in the adherent protein, and HPA is a functional group used to suppress nonspecific protein adsorption, while NHS is used as a functional group to introduce NTA for binding to the His-tagged NTD proteins. After immobilization of NTD on AFM cantilevers, HQ:CSC38/No Al (MikroMasch, Sofia, Bulgaria) with 0.03 N/m spring constant or ATEC-Cont with 0.1–0.3 nN spring constant (Nanosensors, Neuchatel, Switzerland) was used with a DMA/HPA/NHS polymer according to a previous report [18]. Briefly, the cantilever surface was cleaned with 1% HF for 1 min. Subsequently, the cantilever surface was hydroxylated by cleaning with sulfuric peroxide mixture (SPM; 1:1 mixture of H_2_SO_4_ and H_2_O_2_) for 20 min at 80 °C. After rinsing with ultrapure water and ethanol, the cantilevers were completely hydroxylated with a hydrochloric acid–hydrogen peroxide mixture (HPM) with a 6:1:1 ratio of distilled water, HCl, and H_2_O_2_ for 15 min at 80 °C. The cantilevers were rinsed with ultrapure water, ethanol, and 10 mM borate buffer (pH 9.0) prior to polymer modification. The hydroxylated cantilevers were incubated in the polymer solution overnight at 18 °C. The NHS groups were reintroduced into the polymer on the cantilever using a water-soluble carbodiimide (50 mM) in the MES buffer (10 mM, pH 5.0) for 20 min. The cantilever was rinsed with MES and borate buffer before the subsequent modification step. The polymer-coated cantilevers were then treated with 10 mM *N*-(5-Amino-1-carboxypentyl)iminodiacetic acid (AB-NTA, Dojindo Laboratories Co., Ltd., Kumamoto, Japan) to introduce NTA groups via NHS in borate buffer (pH 9.0) for 1 h at 25 °C and subsequently rinsed with borate buffer. The remained NHS groups were blocked with 10 mM ethanolamine HCl in 10 mM borate buffer for 30 min at 25 °C. The cantilevers were then rinsed with borate buffer, followed by distilled water. The Ni^2+^ chelation step with the NTA was performed using 100 mM NiCl_2_ in distilled water for 10 min at 25 °C. His-tagged full-length NTD, GFP-Q1, or GFP-Q4 NTD (20 nM) in PBS (pH 7.5) was immobilized on the polymer-coated cantilevers by incubating for 1 h at 4 °C.

### 2.9. Tensile Test of the Nestin Tail via AFM

Tensile test of the NTD samples was performed using the NTD-modified cantilevers and AFM (CH200; Bruker, Billerica, MA, USA). The cantilever was lowered vertically at a velocity of 1 μm/s and brought into contact with the AF-immobilized substrate until the repulsive force reached 1 nN. The cantilever was then retracted at 1 μm/s to stretch the NTD molecule, and the force curve was recorded until the time that the cantilever returned to its original position. For the repeated tensile test, the NTD-modified cantilever was moved towards the AF with a velocity of 1 μm/s and a set point of 1 nN, and left to dwell for 1 min. The cantilever was then moved 200 nm away from the substrate and moved up and down four times with an amplitude of 300 nm and a velocity of 200 nm/s.

## 3. Results and Discussion

### 3.1. Colocalization of NTD and AFs

**Figure 1:**
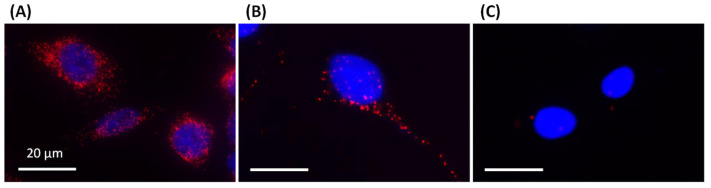
Proximity ligation assay (PLA) for vimentin–actin and nestin–actin. (**A**) Vimentin–actin in the SC2 wild-type cells, (**B**) nestin–actin in the SC2 wild-type cells, and (**C**) nestin–actin in the SC2 nestin knockout cells. The cells were treated with antibodies specific to vimentin, nestin, and actin. The nuclei were stained with DAPI (blue). The PLA foci are indicated in red.

The results of PLA analysis between nestin and AFs are shown in Figure 1. The red fluorescent foci indicated that the two proteins are within 40 nm of each other, suggesting protein binding. Vimentin binds to AFs via the vimentin tail domain (VTD) [14], which we confirmed via PLA (Figure 1), as also shown in a previous report [11]. Fluorescent foci were also observed for nestin and AF in the mouse breast cancer cell SC2, although the number of foci was reduced compared to that with vimentin (Figure 1). If nestin binds to AFs, as suggested by the PLA results, the NTD is a strong candidate for the binding site since the antibodies used in the PLA analysis specifically recognize the NTD. Based on our observation that IF–AF binding contributes to cell stiffness [11], nestin–AF binding may also contribute to form a rigid cytoskeletal network. However, if nestin binds to AFs at the very end of its 170 kDa NTD and the NTD is easily stretched, it is possible that this would form a highly mobile cytoskeleton, lowering cell stiffness. In the mutant rescue of a nestin knockout cell reported in our previous study [11], the cells recovered their flexibility when rescued with the full-length nestin gene. In contrast, cells remained stiff and failed to regain flexibility when rescued using a nestin gene lacking the NTD. These observations suggest that the AF binding site resides on the NTD.

**Figure 2:**
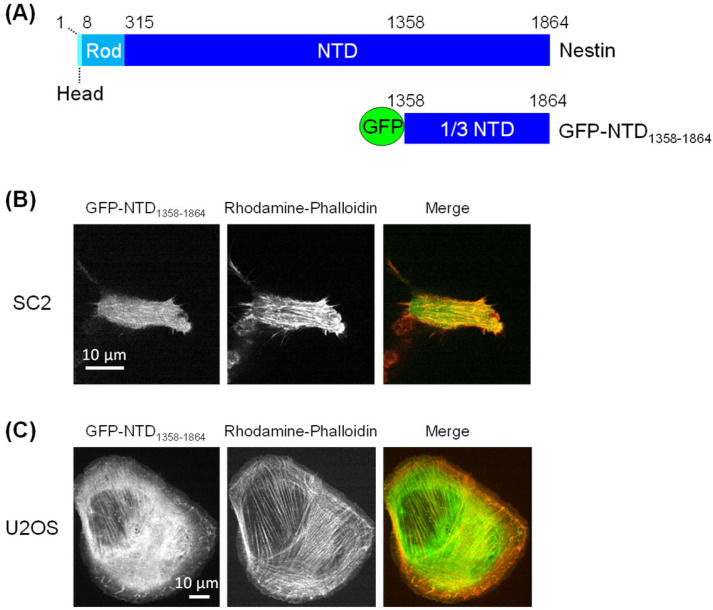
GFP-NTD and AF intracellular colocalization. (**A**) Organization of nestin and GFP fused with NTD_1358–1864_ expressed in cells. Fluorescence microscopy images of SC2 (**B**) and U2OS cells (**C**) expressing GFP fused with NTD_1358–1864_ (green). AFs were stained with rhodamine–phalloidin (red).

We therefore first examined whether the NTD binds to AFs in cells by expressing the GFP-NTD_1358–1864_ fusion protein (Figure 2A). Since the NTD is huge, we used the C-terminal one-third fragment of the NTD in this experiment. As shown in Figure 2B, AF and GFP-NTD_1358–1864_ colocalized in SC2 cells, which was particularly clear on the stress fibers. This result suggests that nestin binds to AFs in the cell with the C-terminal one-third fragment of the NTD. Although the NTD was generated using a mouse-derived gene, colocalization was also confirmed in the human osteosarcoma U2OS line (Figure 2C). It is thought that the mouse NTD is able to bind human AFs since the actin sequence and structure are highly conserved across species.

### 3.2. Direct Interaction Between the NTD and AFs

**Figure 3:**
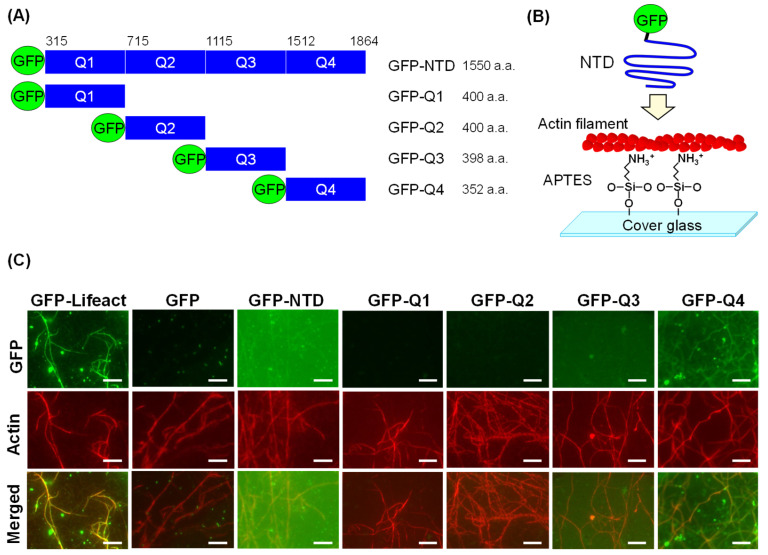
In vitro colocalization assay between NTD and AFs. (**A**) Organization of GFP fused with full-length NTD and truncated mutants Q1 to Q4. (**B**) Scheme of the interaction between GFP-NTD and an AF immobilized on a glass substrate. (**C**) Fluorescence images of GFP, rhodamine–phalloidin-stained AFs, and the merged images. Scale bars: 5 µm.

Next, in vitro experiments were conducted to confirm that the NTD and AFs bind directly. GFP fused to the N-terminus of the NTD (Figure 3A) interacted with AFs immobilized on a glass substrate modified by APTES (Figure 3B). Green fluorescence derived from GFP-Lifeact, which is known to bind to AFs [19], colocalized with the AFs stained with rhodamine–phalloidin (Figure 3C). GFP-NTD also colocalized with the AFs. GFP alone showed no colocalization with AFs. These results indicate that the NTD interacts directly with AFs.

To identify the actin-binding region, we divided the NTD into four parts, Q1–Q4, and produced truncated proteins with GFP fused to the N-terminus. GFP-Q1, -Q2, and -Q3 NTDs did not colocalize with AFs (Figure 3C). In GFP-Q4, green fluorescence of the same shape as the AF was clearly observed. The ratio of the intensity of green fluorescence on the AF to the background was calculated. The signal/noise ratio after washing with high-salt actin polymerization buffer was significantly higher in GFP-Q4 than in GFP-Q1 to -Q3 (Figure S1). This result indicates that the NTD derivatives were nonspecifically adsorbed onto the substrates through electrostatic interaction between APTES and acidic NTDs and when they were washed away with the high-salt actin polymerization buffer from the substrates. The fluorescence of GFP-Q4 on the AFs clearly remained after washing, indicating that GFP-Q4 specifically bound to the AFs. This result, which confirmed the colocalization of GFP-Q4 and AFs even after washing with the high-salt buffer, also suggests that NTD–AF binding may be as stable as antigen–antibody binding [20].

### 3.3. Evaluation of the Mechanical Properties of the NTD via a Tensile Test

**Figure 4:**
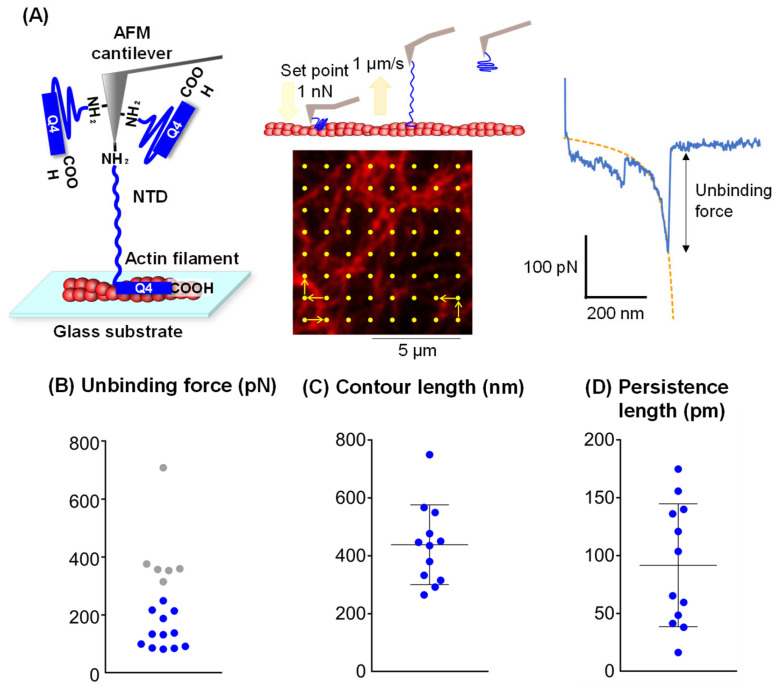
Tensile test of NTD using AFM. (**A**) Scheme of the tensile test using an AFM cantilever with the N-terminus of the immobilized NTD interacting with an AF on a substrate. The yellow dots in the fluorescent image of the AFs indicate the point where the AFM probe was in contact. (**B**) Plot of unbinding force measured from the force curves. The values over 300 pN are shown in gray. (**C**) Plot of contour lengths calculated by the WLC model. The results are presented as means ± standard deviations. (**D**) Plot of persistence length calculated by the WLC model. The results are presented as mean ± standard deviation.

To investigate the interaction between the NTD and AFs, we analyzed the force curve obtained when an AFM cantilever with the immobilized N-terminus of NTD interacted with an AF on a substrate and was pulled apart by the cantilever (Figure 4A). Because the force curves showing characteristic peaks obtained by polymer elongation were obtained at the location of the AFs, these force curves were fitted with the worm-like chain (WLC) model (a model for linear polymers). The force curve with two fitting curves represented the extension of two or more molecules, and the unbinding force was calculated from the force curves excluding these curves (Figure S2). Because the unbinding force between the His-tag and Ni-NTA has been reported to be approximately 150 pN [21], curves with an unbinding force of 300 pN or higher may involve more than two molecules of the NTD stretch, and they were also excluded from the subsequent analysis (Figure 4B). It is not possible to discriminate between dissociation of the His-tag from Ni-NTA and a dissociation of the NTD from AFs. However, NTD–AF binding was highly stable, as suggested by the results shown in Figure S1, suggesting that unbinding of the His-tag from Ni-NTA is more probable. The stable binding of NTD to AF is reasonable in terms of maintaining the three-dimensional structure of the cytoskeletal network [1].

The force curves obtained from the NTD tensile tests were fitted using the WLC model. The average contour length was 438 nm (Figure 4C). Assuming that the NTD was fully extended in a manner similar to the β strand structure, the maximum length of the NTD of 1550 residues is approximately 543 nm, given that the distance between alpha carbons in the same orientation within the idealized β strand is 0.7 nm [22]. This average contour length corresponded to approximately 80% of the maximum NTD length, indicating that the NTD had a highly extensible structure. The maximum length between the N-terminus of the NTD and residue 1240 was 434 nm, indicating that the NTD binds to the AF after residue 1240. This is consistent with our domain analysis, which showed that Q4, starting at residue 1199, exhibits actin-binding activity. The average persistence length of the extended NTD, which represents the rigidity of the molecule, was 84 pm, as estimated by the WLC model (Figure 4D). Compared with previously reported persistence lengths of several hundred picometers for other proteins, such as tenascin [23] and spectrin [24], the persistence length of the NTD was small. These results suggest that the NTD is a flexible and soft molecule (i.e., it can be greatly extended by a weak force). When the NTD molecule is pulled, no kink appears in the curve, suggesting that NTD is gradually stretched to be a beta structure [25,26].

Additional tensile tests were performed using an AFM tip modified with GFP-Q1 or GFP-Q4 and an AF substrate. We did not analyze molecular extension from the force curve, but instead focused on the occurrence of an unbinding force. In experiments using GFP-Q4, unbinding forces exceeding 100 pN were repeatedly detected, with a frequency of occurrence at 12.1%. In contrast, these high unbinding forces were rarely observed with GFP-Q1NTD, with a detection frequency of only 1.8%. This result also suggests the presence of an AF binding site within Q4NTD.

### 3.4. Repeated Tensile Test for NTD

**Figure 5:**
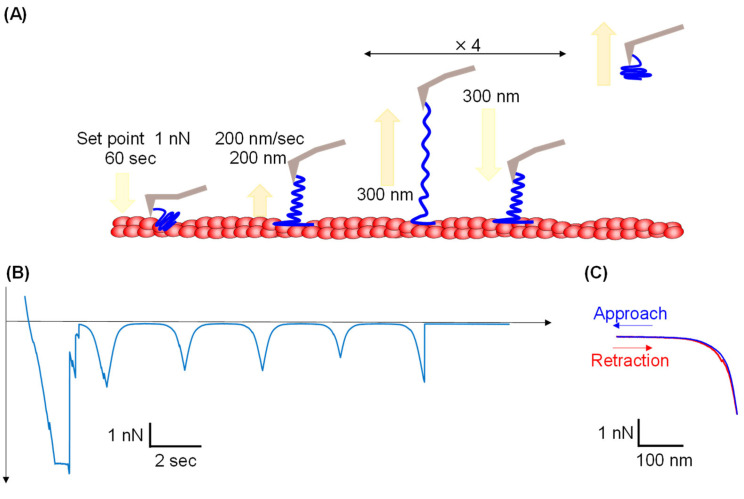
Repeated tensile test of the NTD using AFM. (**A**) Scheme of repeated tensile test for the NTD. Left/right arrow indicates processes that operated four times repeatedly. (**B**) Force–time curve obtained by the repeated tensile test for NTD. (**C**) Force–elongation curve obtained by the repeated tensile test for NTD.

Repeated tensile tests were performed to estimate the reversibility of NTD stretching. After allowing the NTD-modified cantilever to contact an AF on the substrate, the cantilever was lifted by 200 nm, and the operation of moving the cantilever up and down was repeated four times for elongation (Figure 5A). When the cantilever was pulled up after the first contact of the AFM tip with the substrate, the first force appeared that represents not only the unbinding force between the many NTDs and the AFs but also a nonspecific interaction between the cantilever and the substrate in the force–time curve, resulting in a large force peak (Figure 5B). Once the cantilever was pulled up, the tip was no longer in direct contact with the substrate, and a single NTD molecule was stretched and relaxed repeatedly. Because it took 20 s to complete the measurement, as shown in the graph, this result also indicates the long binding lifetime of the NTD and AFs.

Symmetric force curves appeared after the first peak, indicating extension and contraction of the NTD during four repetitive up and down movements. All peaks showed that the retraction process correctly traced the extension process, suggesting that the NTD can be repeatedly unfolded and refolded. Therefore, we believe that the unfolding and refolding of the NTD is a reversible process. These peaks were converted to force–elongation curves. Hysteresis was defined as the energy lost in the molecule between loading and unloading. The repeated mechanical stretch for the small protein NuG2 has shown large hysteresis in the unfolding and refolding force curves, indicating that the process was not in equilibrium [26]. As shown in Figure 4C, the hysteresis of NTD unfolding and refolding was small. Even when the NTD was extended over 200 nm, energy dissipation was small, and contraction of the NTD occurred elastically, potentially behaving like a soft spring within the cytoskeleton. Previous studies have predicted that nestin weakens the cytoskeleton and makes cells more flexible by inhibiting the binding of vimentin to AFs [11]. Herein, we confirmed that nestin itself stably binds to AFs at the C-terminus of the NTD, and the NTD is stretched significantly with a weak force. Presumably, this does not impede cellular softening. Additionally, these results suggest that NTD binding to actin contributes to recovering the cytoskeleton to its original organization after mechanical deformation. When the cytoskeleton is mechanically stressed by cell movement, such as cell migration, the IF network is subjected to large loads. Interactions connecting the cytoskeleton are also stressed; however, nestin would absorb the load by extending it with a weak force. In addition, stable binding to AFs would contribute to maintaining the IF network at its original location near the cell membrane, where the AFs exist.

**Figure 6:**
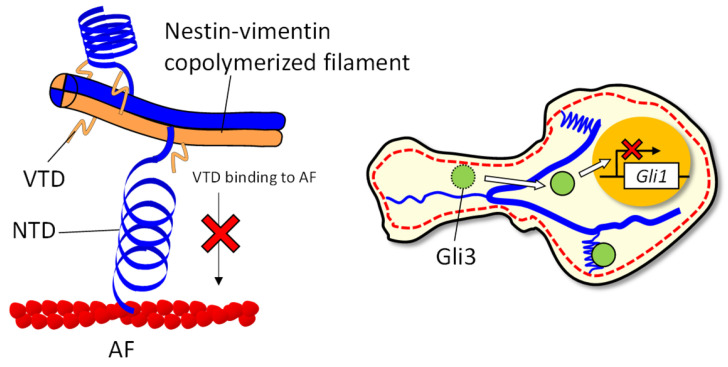
Schematic diagram of the flexible IFs formed by nestin and the release of Gli3 by extension of the NTD.

Another possible consequence of extending the NTD is the release of proteins trapped by nestin. In other IFs, binding of transcription-related proteins has been reported, including PHB2 bound to vimentin [27] and heteronuclear ribonucleoprotein K bound to keratin-19 [28]. Since the binding of proteins, such as Cdk5 [29] and Gli3 [6], to the NTD has been reported, it is natural to assume that bound proteins are released during the complete extension of the NTD. Although speculative, Figure 6 provides a schematic hypothesis based on the findings of this study, including the inhibition of VTD binding to AF. For example, released Gli3 may be transported to the nucleus to inhibit cancer growth signaling. As discussed above, the fact that the NTD is extended by a weak force implies a variety of accompanying functions. There may be structures in the NTD that store elastic energy by unfolding, like talin rod domains [30], warranting more detailed analyses in the future.

## Data Availability

The original contributions presented in the study are included in the article/Supplementary Material; further inquiries can be directed to the corresponding author.

## Supplementary Materials

The following supporting information can be downloaded at https://www.mdpi.com/article/10.3390/cells14020138/s1, Figure S1: Ratio of the GFP fluorescence intensity in AF and background obtained from fluorescent images of an in vitro colocalization assay of AF and GFP-Q1 to -Q4 NTD shown in Figure 3. The intensity was measured before and after washing with high-salt actin polymerization buffer. * *p* < 0.01, n.s. not significant; Figure S2: Example of the force curve obtained by tensile testing of the NTD. Two fitting curves from the WLC model are shown in red and green dashed lines.

## Abbreviations

AF	actin filament
NTD	nestin tail domain
IF	intermediate filament
PLA	proximity ligation assay
NTA	nitrilotriacetic acid
APTES	aminopropyltriethoxysilane
BSA	bovine serum albumin
DMA	dopamine methacrylamide
HPA	2-hydroxypropyl acrylamide
NHS	*N*-hydroxysuccinimide
AFM	atomic force microscopy
VTD	vimentin tail domain
