# Supplementary material for: Nestin Forms a Flexible Cytoskeleton by Means of a Huge Tail Domain That Is Reversibly Stretched and Contracted by Weak Forces
Source: Cells. 2025 Jan 17;14(2):138. doi: 10.3390/cells14020138 (PMC11763517; doi:10.3390/cells14020138)
Supplement: Supplementary file 1 [file cells-14-00138-s001.zip › cells-3337307-supplementary.pdf]

Supplementary Materials

Nestin Forms a Flexible Cytoskeleton by Means of a Huge Tail Domain That Is Reversibly Stretched and Contracted by Weak Forces

Ayana Yamagishi, Rina Tokuoka, Kazuki Imai, Moe Susaki, Mei Mizusawa, Koki Uchida, Saku T. Kijima, Akira Nagasaki, Daijiro Takeshita, Chiaki Yoshikawa, Taro Q. P. Uyeda and Chikashi Nakamura

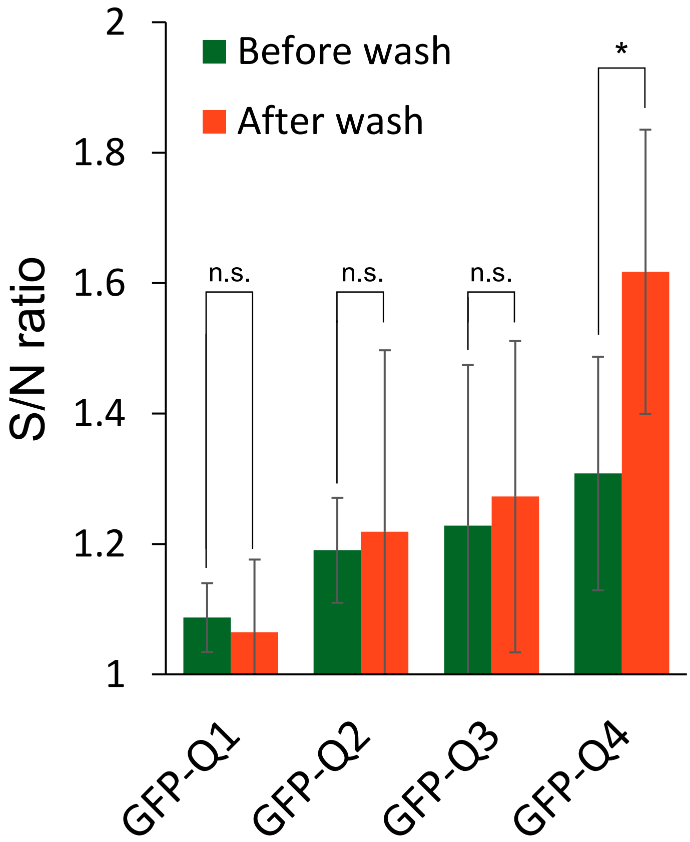

Figure S1. Ratio of the GFP fluorescence intensity in AF and background obtained from fluorescent images of an *in vitro* colocalization assay of AF and GFP-Q1 to Q4 NTD shown in Fig. 3. The intensity was measured before and after washing with high-salt actin polymerization buffer. *p < 0.01, n.s. not significant.

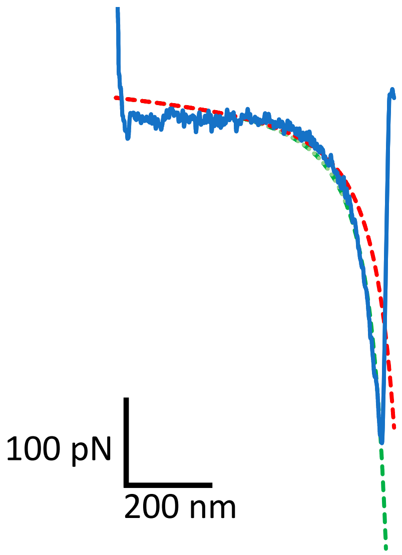

Figure S2. Example of the force curve obtained by tensile testing of the NTD. Two fitting curves from the WLC model are shown in red and green dashed lines.
